# Quantification of 25-hydroxyvitamin D _3_ in dried blood spots as compared to plasma among Indian adults

**DOI:** 10.12688/f1000research.149829.2

**Published:** 2025-06-06

**Authors:** Ashwini V Shete, Jyoti Sawant, Rajani Bagul, Ujjwala Ghule, Sarang S. Saluke, Christopher R. Sudfeld

**Affiliations:** 1 ICMR National AIDS Research Institute, Pune, Maharashtra, India; 2 Bio-Analytical Technologies, Pune, Maharashtra, India; 3 Global Health and Population, Harvard T.H. Chan School of Public Health, Boston, USA

**Keywords:** Vitamin D, Calcifediol, Micronutrients, Dried Blood Spot Testing, Validation Study

## Abstract

**Background:**

Vitamin D may play an important role in later-life physical and cognitive health. Vitamin D status is standardly assessed in serum and plasma; however, collection, transport, and storage costs make large epidemiologic studies challenging. We assessed the agreement of 25-hydroxyvitamin D _3_ (25(OH)D _3_) quantification from dried blood spots (DBS) as compared to standard plasma assessment among older Indian adults.

**Methods:**

A total of 58 adults over 45 years of age who resided in Pune, India were enrolled in the study from July 2020 to June 2021. Liquid chromatography-tandem mass spectrometry (LC-MS/MS) was used to assess 25(OH)D _3_ concentrations in paired plasma and DBS samples.

**Results:**

Plasma and DBS 25(OH)D _3_ concentrations were highly correlated (Pearson’s correlation = 0.976). The median 25(OH)D _3_ concentration of the study population assessed by plasma was 14.6 ng/mL (Q1=12.0, Q3= 18.1) while the median concentration assessed in DBS was 12.8 ng/mL (Q1=11.0, Q3= 16.6). 25(OH)D _3_ concentrations measured from DBS were on average 6% (95% CI: 2-13%) lower than concentrations assessed by plasma across the observed 25(OH)D _3_ distribution.

**Conclusions:**

We found good agreement between 25(OH)D _3_ quantification between DBS and plasma and our findings indicate that DBS can be used in epidemiologic studies of vitamin D among Indian adults.

## Introduction

Globally, it is estimated that one billion people are vitamin D deficient (25(OH) D <20 ng/mL), while almost 50% of the global population is estimated to be vitamin D insufficient (25(OH) D 20-30 ng/mL). ^1^ Sun exposure and dietary sources of vitamin D (e.g. fatty fish, milk, cheese, vitamin supplements) contribute to vitamin D status. ^2^ Older adults may be at greater risk of vitamin D deficiency due to decreased production of vitamin D in the skin, reductions in sun exposure and physical activity, decreased appetite, and changes in dietary habits. ^3^ In India, studies have indicated that vitamin D deficiency is common among the general adult population; however, few studies have included population-based samples. ^4^

Vitamin D status in humans is best indicated by 25-hydroxyvitamin D (25(OH)D), which is the total of the 25-hydroxy forms of 25-hydroxyvitamin D _2_ (25(OH)D _2_) and 25-hydroxyvitamin D _3_ (25(OH)D _3_). ^5^ Serum or plasma quantification of 25(OH) D are the standard biological specimens used in the assessment of vitamin D status. ^6^ However, in large epidemiologic studies, collection and storage of plasma or serum are often not feasible due to the cost and logistic requirements of refrigeration after blood collection and cryogenic storage, particularly in resource-limited settings. As a result, dried blood spot (DBS) collection is becoming more common in epidemiologic studies to overcome these barriers. Prior studies have found that measurement of 25(OH) D from DBS with liquid chromatography-tandem mass spectrometry (LC-MS/MS) can yield valid and reliable results. ^7^^–^^13^ However, older age may alter vitamin D binding protein levels and affinity and influence vitamin D level estimations in the elderly population. ^14^

We conducted a study to evaluate the agreement of 25(OH)D _3_ assessment in DBS as compared to plasma concentrations among Indian adults over 45 years of age. The results of our study are intended to inform the ability to use DBS to assess vitamin D status in large population-based studies of vitamin D status among older adult populations in India.

## Methods

### Study design

The study enrolled 58 adult participants over 45 years of age who resided in Pune, India. Participants were contacted for enrollment at ICMR-NARI Facility for Collaborative Clinical Research. The study enrolled participants from July 2020 to June 2021.

### Sample collection

Each participant had 4 mL of blood collected with a lavender top vacutainer (ethylenediaminetetraacetic acid (EDTA) anticoagulant). 50 μl of whole blood was spotted on predefined circles of Whatman 903 filter paper using a calibrated pipette. The DBS were air-dried and then stored at -20°C by placing them individually into a zip lock bag along with desiccants and a humidity indicator card. The blood sample was also centrifuged at 1500 g for 15 minutes to isolate plasma. The plasma samples were stored at -80°C until testing.

### Chemicals and reagents

The study utilized D6-25-hydroxyvitamin D3 (IS) and 25-hydroxyvitamin D3 (25(OH)D3) from Sigma Aldrich (St. Louis, USA, catalog numbers H-074-1ML and 17938-1MG, respectively), HPLC grade methanol from J. T Bakers (Phillipsburg, NJ, USA, catalog number 8402.2500), formic acid from Sigma Aldrich (St. Louis, USA, catalog number F0507-100ML), acetonitrile from J. T Bakers (Phillipsburg, NJ, USA, catalog number 02-002-180), ammonium formate from Loba Chemie (Mumbai, India, catalog number 01210), and zinc sulfate pentahydrate from Thomas Baker (Mumbai, India, catalog number 169722). A set of 5 control samples with known target values of 25(OH)D _3_ were obtained from the DEQAS Vitamin D External Quality Assessment Scheme (London, UK, no catalog number).

### Standard preparation and calibration

A stock solution of the standard, 25-hydroxycholecalciferol (25(OH)D _3_), was prepared in ethanol at a concentration of 1 mg/mL. The standard was diluted to prepare liquid calibrators of concentrations of 5, 10, 20, 40, 60, 80, & 125 ng/mL and controls with the concentrations of 25, 50, and 100 ng/mL using 0.1%formic acid in methanol: water (80:20 v/v) as a diluent. DBS calibrators were prepared in artificial blood consisting of washed RBCs and phosphate buffer pH 6.5 (in water) mixed in 1:1 proportion. 50 μl of the spiked blood was spotted on Whatman filter paper number 903 to prepare standards of concentrations of 5, 10, 20, 40, 60,80, and 125 ng/mL. Controls were prepared similarly with concentrations of 25, 50, and 100 ng/mL.

### LC-MS/MS quantification of 25(OH)D _3_ in plasma

The procedure for measuring 25(OH)D _3_ in plasma samples was as follows: 100 μL internal standard (50 ng in 100% methanol) was added to 100 μL of plasma samples and vortexed for 5 minutes. This was followed by the addition of 50 μL zinc sulfate pentahydrate (0.2 M) and 300 μL acetonitrile: methanol (70:30) with vortexing steps in between. The tubes were centrifuged for 10 minutes at 14000 rpm at 10°C. 200 μL supernatant was transferred to HPLC vials for analysis.

### Sample preparation/extraction procedure for DBS

Two 6mm DBS punches were placed in Eppendorf tubes, to which 100 μL internal standard (50 ng in 100% methanol) was added and vortexed for 5 minutes. Thereafter 50 μL of zinc sulfate pentahydrate (0.2 M) and 300 μL acetonitrile: methanol (70:30) were added sequentially with vortexing steps after each addition. The tubes were centrifuged for 10 minutes at 14000rpm at 10°C and 200 μL supernatant was transferred to an HPLC vial for further analysis.

### LC-MS/MS conditions

The system consisted of 4000 QTrap (A B Sciex) along with the Shimadzu LC 20AD LC System (Shimadzu Corporation, Singapore, no catalog number) with electrospray ionization (ESI) controlled by Analyst 1.4.2 software (SCIEX, MA, USA, https://sciex.com/products/software/analyst-software , no catalog number). Copyright license was obtained for the Analyst 1.4.2 software. The following HPLC conditions were used:

1. Column: Agilent Poroshell 120 EC-C18, (2.1×50 mm, 2.7-Micron particle size)
2. Column temperature: 40°C
3. Injection volume: 10 μl
4. The mobile phase: A: Methanol and B: 10 mM Ammonium Formate in HPLC grade Water
5. The mobile phase composition: A: 90% and B: 10%. Isocratic flow.
6. Flow rate: 0.4 mL/min
7. Sample temperature: 5°C

### Validation of quantitation of 25(OH)D3 by LC-MS/MS method

The LC-MS/MS assay method was validated as per the guidelines for the bioanalytical method development and validation as well as CLSI C62 Liquid Chromatography-Mass Spectrometry Methods; Approved Guidelines. ^15^^,^^16^ The LOQ (limit of quantitation) was set at 5 times the concentration of the LOD (Lower limit of Detection). The LOD was determined by the lowest concentration and its peak-to-peak signal to noise ratio, which was required to be greater than 5. Linearity was determined by analyzing the standard graph for 25(OH)D _3_. Accuracy was determined by comparing the expected versus observed values for the in-house prepared control samples. The control samples were assayed on 5 consecutive days with 6 replicates per day for calculating inter-assay and intra-assay precision, respectively. Recovery was determined by spiking known amounts (5 and 100 ng/mL) of 25(OH)D _3_ in DBS samples, and spiked concentration areas were compared with spiked blank extracted samples. Processed sample stability was assessed after storing the control samples on an autosampler tray at 5°C or benchtop at ambient temperature for 24 hours. Carryover was determined by analyzing the blank sample after the highest standard was measured.

### Statistical analysis

Linearity for plasma quantification and medians and interquartile ranges (IQRs) were presented for 25(OH)D _3_ concentrations for paired plasma and DBS assessments. A Pearson’s correlation was calculated and a Passing and Bablock analysis was conducted to compare plasma and DBS measurements. A Bland-Altman analysis was also conducted to estimate the mean bias and limits of agreement. Analyses were conducted with SAS version 9.3 and MedCalc version 20.027.

## Results

Plasma 25(OH)D _3_ quantitation by LC-MS/MS method was validated first before proceeding to DBS validation. Retention time for 25(OH)D _3_ was observed to be 1.14 minutes. Plasma standards were linear between 5.0 to 125.0 ng/mL with r ^2^ = 0.9988. Accuracy for the controls ranged from 95.02 to 101.3% and intra-assay precision varied from 5.0-6.4%. Vitamin D EQAS samples processed by LC-MS/MS method showed bias ranging from -3.32 to 3.26%.

**Figure 1.**
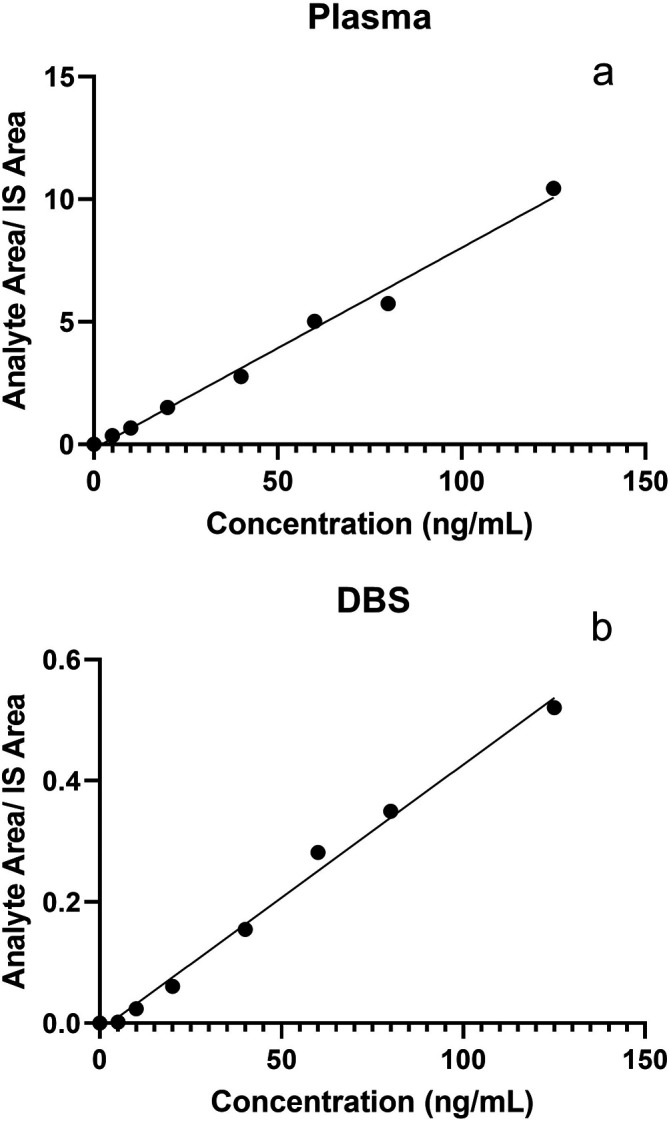
Standard calibration curves for 25(OH)D _3_ concentrations (ng/mL) (x-axis) and Analyte area/IS area ratio (y-axis) in plasma (a) and (b) dried blood spot (DBS) samples.

We then performed LC-MS/MS method validation on DBS samples that were spiked with known 25(OH)D _3_ concentrations in artificial blood. The comparative results of plasma and DBS sample validation are presented in Table 1. DBS 25(OH)D _3_ standards showed good linearity over the working range from 5.0 to 125.0 ng/mL (r ^2^ = 0.9967). Calibration curves for plasma and DBS standards are plotted in Figure 1. The peak-to-peak signal-to-noise ratio for 1 ng/mL standard was ~7. So LOD for the method was 1 ng/mL and the LOQ was 5 ng/mL. Accuracy for the analyte ranged from 100.50% to 113.75%. Intra-assay CVs varied from 5.13% to 13.39% and inter-assay CVs were 6.92% to 13.77% when measured using the in-house prepared QC samples. Recovery of 25(OH)D _3_ varied from 78.11-82.85% while the recovery of the internal standard was up to 90%. Retention time for 25(OH)D _3_ was observed to be 1.14 minutes. 25(OH)D _3_ was found to be stable after the QC samples were processed and left either on the autosampler tray at 5°C or benchtop at ambient temperature for 24 hours. No carryover was observed in the LCMS/MS analysis at a concentration of 100 ng/mL.

**Table 1.** Results of LC MS/MS method validation for 25(OH)D _3_ quantitation using plasma and DBS standards/controls spiked with known 25(OH)D _3_ concentrations.

Parameters	Plasma	Dried Blood Spot (DBS)
Linearity (R ^2^)	0.9988	0.9967
Linearity range	5 to 125 ng/mL	5 to 125 ng/mL
Limit of detection	1 ng/mL	1 ng/mL
Limit of quantification	5 ng/mL	5 ng/mL

Concentrations	5 ng/mL	25 ng/mL	100 ng/mL	5 ng/mL	25 ng/mL	100 ng/mL
Accuracy (%)	95.02	97.588	101.29	100.50	113.75	103.17
Inter-assay precision (CV%)	0.21	3.96	9.51	13.39	9.05	5.13
Intra-assay precision (CV%)	6.42	5.01	6.06	6.92	8.82	13.77
Recovery (%)	-	-	-	82.84	-	78.11
Processed sample stability (%) on Autosampler	-	-	-	93.12	-	99.33
Processed sample stability (%) on Bench Top	-	-	-	100.01	-	102.30

We then proceeded to compare human plasma and DBS samples from study participants. A total of 58 adults participated in the study of which 30 (51.7%) were male and 28 (48.3%) were female. The median age of study participants was 52 years (Q1 = 47, Q3 = 55). The median plasma 25(OH)D _3_ concentration of the study population was 14.6 ng/mL (Q1 = 12.0, Q3 = 18.1, range = 8.0-33.6) while the median DBS 25(OH)D _3_ concentration was 12.8 ng/mL (Q1 = 11.0, Q3 = 16.6, range = 7.7-31.9). Plasma and DBS 25(OH)D _3_ concentrations were highly correlated with a Pearson’s correlation of 0.976 (p < 0.0001).

**Figure 2.**
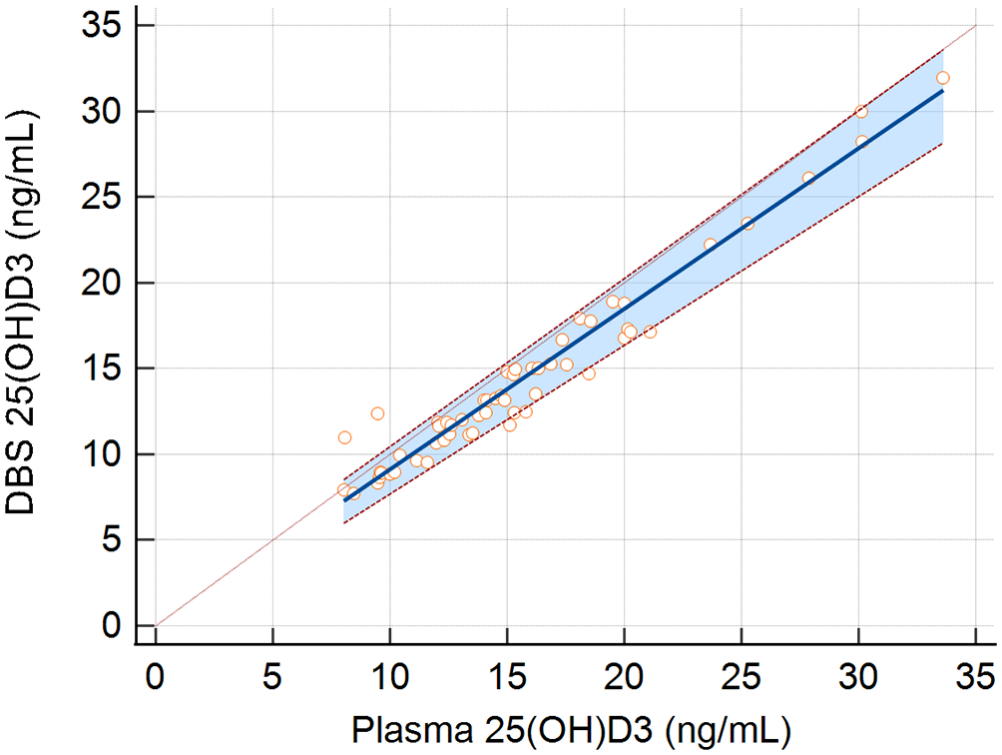
25(OH)D _3_ concentrations (ng/mL) in plasma (x-axis) and in dried blood spots from human samples (DBS; y-axis). Blue line indicates the Passing and Bablok regression equation with red dashed lines and shading indicating 95% confidence intervals. The identity line is presented as a green dashed line.

**Figure 3.**
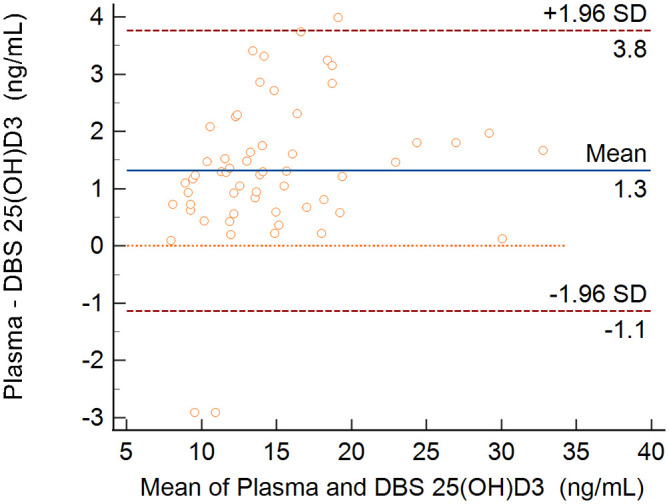
Bland-Altman plot for 25(OH)D _3_ assessment in plasma and in dried blood spots. Difference in concentrations between plasma and DBS (y-axis) plotted against mean of plasma and DBS measurements (x-axis).

Figure 2 presents a plot of the plasma and DBS 25(OH)D _3_ concentrations of study participants. The Passing and Bablock equation for the relationship between DBS and plasma concentrations in the study population was DBS 25(OH)D _3_ concentration (ng/mL) = -0.23 (95% CI: -0.98 to 0.66) + 0.94 × plasma 25(OH)D _3_ concentration (ng/mL) (95% CI: 0.87 to 0.98). The corresponding equation to estimate plasma 25(OH)D _3_ is plasma 25(OH)D _3_ (ng/mL) = 1.0638 * [DBS 25(OH)D _3_ concentration (ng/mL) + 0.23]. There was no indication of non-linearity (p-value = 0.20). Therefore, 25(OH)D _3_ concentrations as assessed by DBS were on average 6% (95% CI: 2-13%) lower than concentrations assessed in plasma across the observed 25(OH)D _3_ distribution. Figure 3 presents the Bland-Altman plot. The mean bias ± standard error between plasma and DBS was 1.31 ± 0.16 ng/mL, and the limits of agreement were -1.14 to + 3.77 ng/mL.

## Discussion

Our study indicated satisfactory performance characteristics for the assessment of 25(OH)D _3_ in plasma and DBS samples using standards and spiked controls. Subsequently, among samples from Indian adults older than 45 years of age, we found a good agreement of measurement of 25(OH)D _3_ from DBS and plasma samples. We found that 25(OH)D _3_ concentrations in DBS samples were on average 6% (95% CI: 2-13%) lower than paired plasma samples. The difference was consistent across the observed range of 25(OH)D _3_ in plasma from 8-32 ng/mL.

Our finding that 25(OH)D _3_ quantification in dried blood spots is a valid alternative to standard plasma assessment is in line with prior validation studies in other settings and populations. ^7^^–^^12^ Further, most prior studies have also found that 25(OH)D _3_ assessed in DBS is systematically lower than serum or plasma concentrations. ^7^^,^^9^^,^^11^^,^^12^ A study conducted among adults 22-48 years in India found that mean 25(OH)D _3_ concentrations were similar between DBS and serum, and there was no difference in the classification of vitamin D sufficiency and insufficiency. ^9^

The study had several limitations. First, we did not quantify 25(OH)D _2_ concentrations in the study population. Nevertheless, it is estimated that 25(OH)D _3_ accounts for the majority of the total 25(OH) D concentrations among adults in India and there is no current large-scale fortification with ergocalciferol (vitamin D _2_) in the country. ^9^^,^^12^ Therefore, the assessment of 25(OH)D _3_ may capture most of the variation in 25(OH) D concentrations in our study population. However, future studies should also evaluate dried blood spot assessment of 25(OH)D _2_ in India to more accurately assess total 25(OH) D. We also saw some evidence of a moderately high intra-assay precision (CV%) for DBS at the 100 ng/mL level, and therefore, future studies may also examine higher ranges of 25(OH)D _3_. Second, we did not adjust for the hematocrit fraction. However, a prior study suggests that adjustment for hematocrit fraction in the normal range had minimal effect on DBS 25(OH)D _3_ quantification. ^13^ Measurement of hematocrit in large population-based studies may not be efficient in terms of resources for the potentially small increase in precision.

Overall, we found good agreement between 25(OH)D _3_ quantification by LC-MS/MS in DBS as compared to standard plasma assessment. DBS assessment of 25(OH)D _3_ may help expand population-based vitamin D research in adult populations in India and other settings.

### Ethics and consent

The study was conducted according to the guidelines of the Declaration of Helsinki and approved by the National AIDS Research Institute Ethics Committee (Ref No. NARI-EC/2017-03, Approval date: 18 August 2020), Indian Council of Medical Research (Ref No. 54/17/Indo-foreign/Ger/17-NCD-II, Approval date: 9 October 2017), and the Harvard T. H. Chan School of Public Health Institutional Review Board (Ref. No. IRB17-0778, 8 June 2017). All participants provided written informed consent.

## Data Availability

Figshare: Vitamin D Validation – India, Sudfeld, Christopher (2024). Vitamin D Validation - India. figshare. Dataset. https://doi.org/10.6084/m9.figshare.25665483.v3. ^17^ This project contains the following underlying data:
•
vitd4222024.csv Data are available under the terms of Creative Commons Zero (CC0) license.

## Software availability

The LC-MS/MS data was analyzed with SCIEX Analyst 1.4.2 software. A free version of software able to complete this analysis is available from Open MS ( https://openms.de/).
