# Supplementary material for: Quantification of 25-hydroxyvitamin D 3 in dried blood spots as compared to plasma among Indian adults
Source: F1000Res. 2025 Jun 6;13:512. Originally published 2024 May 20. [Version 2] doi: 10.12688/f1000research.149829.2 (PMC12895100; doi:10.12688/f1000research.149829.2)
Supplement: Peer review history

# Reviewer report: Reviewer response for version 2

**DOI:** 10.5256/f1000research.182852.r435234

**Published:** 2026-02-11

**Authors:** Y S Phaneendra Mallimoggala

**Affiliations:** 1 Manipal Academy of Higher Education, Manipal, Karnataka, India

Respected Editor,

 The manuscript titled "Quantification of 25-hydroxyvitamin D _3_ in dried blood spots as compared to plasma among Indian adults [version 2; peer review: 1 approved, 1 approved with reservations]" addresses an important analytical topic in the field of laboratory diagnosis.

 A few suggestions to the authors are provided below.

 1. Please provide the chromatogram

 2. Provide an overlay chromatogram showing plasma Vitamin D and DBS Vitamin D from the same volunteer/ calibrator

 3. As a standard protocol, the hematocrit effect should be assessed for DBS; however, the authors have mentioned the lack of this analysis as a limitation.

 4. The authors are requested to provide their view on volcano effect

 5. Please explore the GREEN Chemistry Assessment of this method

 6. Add a note on why DBS DBS-based assessment is needed

 7. Please change the term "Sample temperature" in the LCMS/MS condition subsection of the methodology to "Autosampler temperature"

Is the work clearly and accurately presented and does it cite the current literature?

Yes

If applicable, is the statistical analysis and its interpretation appropriate?

Yes

Are all the source data underlying the results available to ensure full reproducibility?

Partly

Is the study design appropriate and is the work technically sound?

Yes

Are the conclusions drawn adequately supported by the results?

Partly

Are sufficient details of methods and analysis provided to allow replication by others?

Partly

Reviewer Expertise:

Method development and validation, chromatography, inborn errors of metabolism, laboratory diagnostics

I confirm that I have read this submission and believe that I have an appropriate level of expertise to confirm that it is of an acceptable scientific standard, however I have significant reservations, as outlined above.

# Reviewer report: Reviewer response for version 2

**DOI:** 10.5256/f1000research.182852.r390095

**Published:** 2025-06-30

**Authors:** Katarzyna Dzik

**Affiliations:** 1 University of Gdansk, Gdańsk, Poland

The manuscript is now acceptable for indexing.

Is the work clearly and accurately presented and does it cite the current literature?

Yes

If applicable, is the statistical analysis and its interpretation appropriate?

I cannot comment. A qualified statistician is required.

Are all the source data underlying the results available to ensure full reproducibility?

Yes

Is the study design appropriate and is the work technically sound?

Yes

Are the conclusions drawn adequately supported by the results?

Partly

Are sufficient details of methods and analysis provided to allow replication by others?

Yes

Reviewer Expertise:

neurodegeneration, vitamin D, exercise,

I confirm that I have read this submission and believe that I have an appropriate level of expertise to confirm that it is of an acceptable scientific standard.

# Reviewer report: Reviewer response for version 1

**DOI:** 10.5256/f1000research.164335.r312373

**Published:** 2024-09-04

**Authors:** Katarzyna Dzik

**Affiliations:** 1 University of Gdansk, Gdańsk, Poland

The manuscript entitled "Quantification of 25-hydroxyvitamin D _3_ in dried...." is  well written and shows a simple yet important set of data presented in acceptable form.

 I have one major concern.

 In table 1. results show that the accuracy of DBS and plasma is different for 5 and 25 ng/mL concentrations. Also, there is very high CV% for DBS at 5 ng/mL concentration- 13.39. This results might impact the concentration corresponding to vitamin D deficiencies, therefore might be at the great importance. Please address this data in the discussion.

 Minor comments.

 1. Please unify - over — in the description of LC-MS/MS conditions. It might be misleading and seems that the column temperature is minus 40℃.

 2. In the introduction the sentence „Dietary sources of vitamin D (…) are not regularly consumed in most parts of the world and therefore exposure to sunlight is the primary source of vitamin D for most individuals globally.” seems  to be very misleading. The Authors cite a paper by Ann Pretice, however in the original paper by Ann Pretice we can find a sentence „The relative contributions of sunshine exposure and dietary intake to vitamin D supply and the achievement of adequacy in an individual, therefore, depend on many factors.” It would be better to avoid this sentence, or write more information to avoid misleading.

Is the work clearly and accurately presented and does it cite the current literature?

Yes

If applicable, is the statistical analysis and its interpretation appropriate?

I cannot comment. A qualified statistician is required.

Are all the source data underlying the results available to ensure full reproducibility?

Yes

Is the study design appropriate and is the work technically sound?

Yes

Are the conclusions drawn adequately supported by the results?

Partly

Are sufficient details of methods and analysis provided to allow replication by others?

Yes

Reviewer Expertise:

neurodegeneration, vitamin D, exercise,

I confirm that I have read this submission and believe that I have an appropriate level of expertise to confirm that it is of an acceptable scientific standard, however I have significant reservations, as outlined above.

# Response

**Published:** 2025-05-25

**Authors:** Christopher Sudfeld

**Affiliations:** Global Health and Popualtion, Harvard University, Boston, Massachusetts, USA

Thank you for the thoughtful review of the manuscript. We have addressed each of your points below

 Major comment #1 - We agree there was some indication of a potentially higher CV% at 100 ng/mL in the DBS - as a result, we have added this limitation for future studies to look at even higher concentrations. We have noted "We did see some evidence of a moderately high intra-assay precision (CV%) for DBS at the 100 ng/mL level and therefore future studies may also examine higher ranges of 25(OH)D _3_."

 Minor Comment #1 - We have used colon : rather than - to avoid confusion on the temperatures in particular.  

 Minor Comment #2 - We have clarified this sentence to be more general about vitamin D sources. "Sun exposure and dietary sources (e.g., fatty fish, milk, cheese, vitamin supplements) contribute to vitamin D status (2).

# Reviewer report: Reviewer response for version 1

**DOI:** 10.5256/f1000research.164335.r300657

**Published:** 2024-08-13

**Authors:** Christopher T Sempos

**Affiliations:** 1 Vitamin D Standardization Program, Havre de Grace, MD, USA; 2 National Institutes of Health (NIH), Bethesda, MD, USA

Summary: Object is to develop a dried blood spot (DBS) assay for the measurement of plasma 25(OH)D3 concentration that is accurate and precise compared to the Vitamin D Standardization Program (VDSP) standardized LC-MS/MS assay. That is, results from the LC-MS/MS assay can be interpreted as the "True" 25(OH)D concentration.

 Comments:

 (1) P3, Chemicals and Reagents: The authors indicate that the that the LC-MS/MS produced results that were within -5% to +5% of the true 25(OH)D concentration of the DEQAS samples, As a result, the LC-MS/MS assay appears to be standardized according to criteria adopted the Vitamin D Standardization Program (VDSP) -- that is 25(OH)D3 measurements by the LC-MS/MS assay are the true concentration. If the authors reversed X and Y in Figure 2 the resulting regression equation could be used to standardize the DBS values to the true 25(OH)D concentration. I suggest that the make that change and then in the Discussion indicate the importance measuring the True 25(OH)D concentration for public health policy.

 (2) What do the authors mean when they write that "...25(OH)D3 accounts for the majority of the total 25(OH)D among adults in India..."? Even small quantifiable amounts of 25(OH)D2 can make an enormous difference when estimating the prevalence of low/deficient total 25(OH)D levels.

 From the National Indian Survey or other sources what are the 25(OH)D2 concentrations by age and sex.  if this assay is to be used in the study of vitamin D in adults it needs to be established that 25(OH)D2 levels are close to nil in Indian adults.

Is the work clearly and accurately presented and does it cite the current literature?

Yes

If applicable, is the statistical analysis and its interpretation appropriate?

Yes

Are all the source data underlying the results available to ensure full reproducibility?

Yes

Is the study design appropriate and is the work technically sound?

Yes

Are the conclusions drawn adequately supported by the results?

Yes

Are sufficient details of methods and analysis provided to allow replication by others?

Yes

Reviewer Expertise:

Coordinator, Vitamin D Standardization Program

I confirm that I have read this submission and believe that I have an appropriate level of expertise to confirm that it is of an acceptable scientific standard, however I have significant reservations, as outlined above.

## References

[1] : Quantification of 25-hydroxyvitamin D3 in dried blood spots as compared to plasma among Indian adults. *F1000Research* .2024;13: 10.12688/f1000research.149829.1 10.12688/f1000research.149829.1 PMC1289510041695203
